# Analytical and Numerical Investigation of an Electrochemical Chloride Barrier for Reinforced Concrete Structures

**DOI:** 10.3390/ma14216728

**Published:** 2021-11-08

**Authors:** Carla Driessen-Ohlenforst, Michael Raupach

**Affiliations:** Institute of Building Materials Research, RWTH Aachen University, Schinkelstrasse 3, 52062 Aachen, Germany; raupach@ibac.rwth-aachen.de

**Keywords:** preventive cathodic protection, chloride ingress, numerical simulations, carbon-reinforced concrete

## Abstract

During the development of a carbon-reinforced mortar interlayer for bridges, the idea of an electrochemical chloride barrier arose. An electrical field is generated between two carbon meshes, and the negatively charged chloride ions are held on the polarized upper carbon mesh to prevent chloride-induced corrosion in the reinforcement. Laboratory tests unexpectedly showed that higher voltages lead to an increase in chloride ions for certain depths of the reference probes. This paper discusses the implementation of analytical and numerical models that finally explain the effect only by the acting diffusion and migration with the help of a finite differences model and finite elements simulations. The effect of the local minimum is limited to positions above the depth of the first carbon layer of the test specimens. It is caused by the lines of the electrical field between the first and second carbon layer. According to the experimental and finite elements simulation results, higher voltages lead to lower chloride concentrations for all positions below the first carbon layer only after sufficient time duration. Therefore, the intended effect of an electrochemical chloride barrier can in general only be observed and confirmed after a certain time depending on position, conditions and parameters.

## 1. Introduction

In the course of the research project, an interlayer for bridges was developed to be used for multiple purposes. The 35 mm thin interlayer consisted of two carbon meshes with a spacing of 15 mm which were each embedded in a newly developed mortar. By measuring the resistivity between the carbon meshes, leakages in the sealing layer of the bridge were detected by decreasing resistivity values due to intruding water. When a leak was detected, an external voltage between the two carbon layers was supposed to reduce the chloride ingress and, therefore, prevent corrosion damage in the reinforcement. Furthermore, the textile reinforcement led to greater shear force strength [1,2]. The function of chloride ingress prevention was named the “electrochemical chloride barrier”. It aims to reduce or even eliminate the necessity of expensive maintenance and repair measures due to chloride-induced corrosion of the reinforcement. Thus, traffic obstructions, which often lead to economic losses, can be prevented.

To investigate and confirm the effectiveness of the function of chloride ingress prevention, laboratory tests were performed. In [3], it was demonstrated that voltages between 0.5 and 2 V lead to a reduction in chloride concentration behind the anodic polarized mesh for water-saturated specimens and carbon, as well as titanium as an anode material. Due to the destructive testing method of chloride titration on the grinded probes, no time effect was investigated in the laboratory. In order to take any time-dependent effects into account, analytical and numerical investigations were performed which will be described in this paper. Furthermore, the laboratory test results showed that higher voltages do not generally lead to lower chloride concentrations in all specimen depths at the investigated testing time but that certain local minima exist, up to which the concentration decreases and the voltage increases, in order to increase again when a certain voltage value is passed. An investigation of this effect over time and space is focused on throughout the investigations detailed in this paper to clear out potential doubts about the effectiveness of the electrochemical chloride barrier.

**Figure 1:**
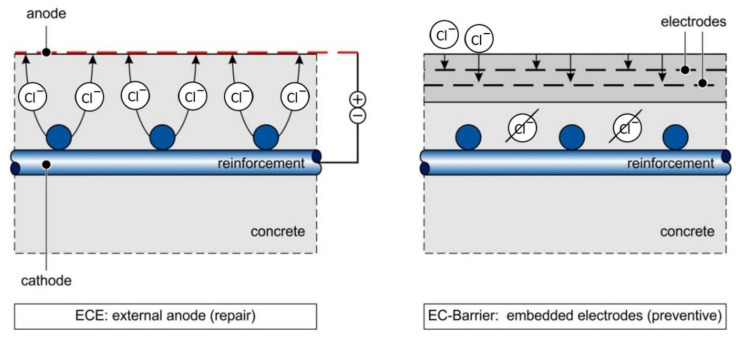
Comparison ECE vs. EC-barrier.

To date, various research works have focused on the question of electrochemical chloride extraction (ECE) [4,5,6]. In these studies, simulations have also been used in order to investigate the effect of chloride movement through concrete; however, in comparison with this work, major differences exist [7,8,9]—firstly, for electrochemical chloride extraction, much higher voltages are common and lower voltage ranges are not usually examined. Secondly, the chloride ions are already in the concrete. Finally, due to the anode system which is placed on top of the structure, the migration is unidirectional from the steel to the anode system; whereas, in the case of the electrochemical barrier, the flux direction changes in the structure depending on which area is observed. The latter is the starting point for the discussion in the following. Figure 1 provides a schematic comparison of the above-mentioned differences between previous work and this paper.

## 2. Materials and Methods

To start any numerical and analytical investigations of the time- and space-dependent behavior of the chloride ions in an EC-barrier (Figure 1), it must be described as a mathematical–physical problem, which is to be solved. A reasonable simplification of the problem and the boundary as well as initial conditions is targeted to also allow for the derivation of analytical and efficient numerical solution approaches to explain the experimentally observed phenomenon.

### 2.1. Simplified Mathematical–Physical Problem

**Figure 2:**
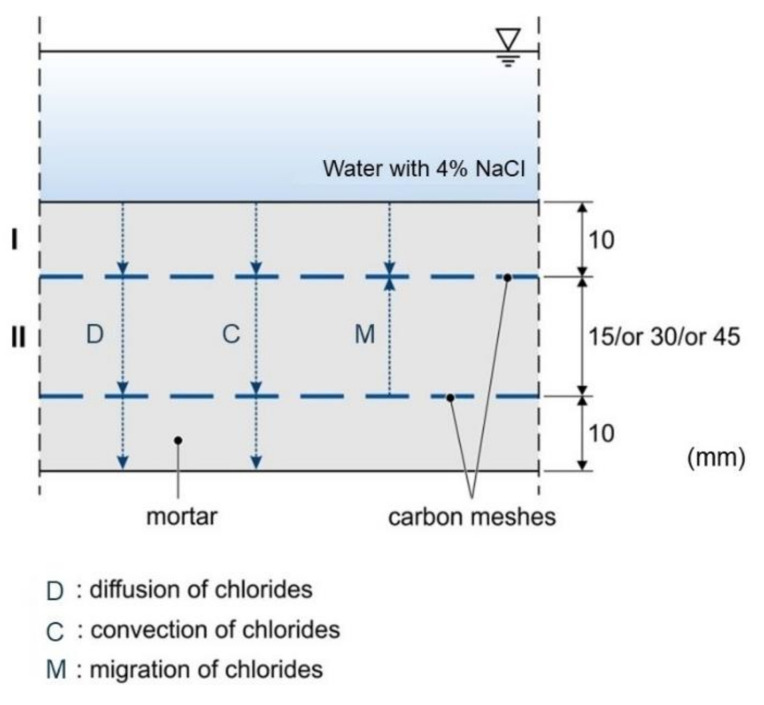
Schematic picture of the investigated structure and ion movement.

The flow of the particles in the probe can be described in the two areas—first, from the top of the specimen to the first carbon layer (I) (see Equation (1)) and, second, from the first to the second carbon layer (II) (see Equation (2)), which can be seen in Figure 2.

There is a diffusion term, which results from Fick’s first law, as well as a convection and migration term originating from the direction of the electrical field and the water-flow into the probe, respectively. The latter are assumed here as constants in time and space. This results from the considerations that the height of the bath—with the 4% NaCl solution that was applied to the top of the probes, and thus the value of the hydrostatic pressure—is constant over time, as well as from the incompressibility of the fluid and a constant electrical field in between the layers.

(1) $$\left(I\right)\;J=-{\;D}_{\mathrm{eff}}\times \frac{\partial c}{\partial x}+v\times c+u_{m}\times c$$

(2) $$\left(\mathrm{II}\right)\;J=-{\;D}_{\mathrm{eff}}\times \frac{\partial c}{\partial x}-v\times c+u_{m}\times c,$$

where:

- J: Molar flow of the NaCl ions (mol/(m² s));
- $D_{\mathrm{eff}}$: Diffusion coefficient of NaCl ions in mortar (m²/s);
- c: Concentration of NaCl ions in mortar (mol/m³);
- x: Position along the depth of the probe (m);
- v: Average speed of NaCl ions in electrical field (m/s);
- $u_{m}$: Average mass-related speed (m/s).

The continuity equation describes the relationship between the change in concentration in volume over time and the flow:

(3) $$\frac{\partial c}{\partial t}+\frac{\partial J}{\partial x}=0$$

after substituting Equations (1) and (2) into Equation (3), we obtain:

(4) $$\left(I\right)\frac{\partial c}{\partial t}={\;D}_{\mathrm{eff}}\times \frac{\partial^{2}c}{\partial x^{2}}-\left(v+u_{m}\right)\times \frac{\partial c}{\partial x}$$

(5) $$\left(\mathrm{II}\right)\frac{\partial c}{\partial t}={\;D}_{\mathrm{eff}}\times \frac{\partial^{2}c}{\partial x^{2}}-\left(u_{m}-v\right)\times \frac{\partial c}{\partial x}$$

by combining the constants, v and $u_{m}$, both equations can be represented in the familiar form of the one-dimensional unsteady convection–diffusion equation:

(6) $$\frac{\partial c}{\partial t}={\;D}_{\mathrm{eff}}\times \frac{\partial^{2}c}{\partial x^{2}}-v_{\mathrm{eff}}\times \frac{\partial c}{\partial x},$$

where:

(7) $$\left(I\right){\;v}_{\mathrm{eff}}=v+u_{m}$$

(8) $$\left(\mathrm{II}\right){\;v}_{\mathrm{eff}}=u_{m}-v,$$

where $v_{\mathrm{eff}}$: Effective speed of the NaCl ions (m/s).

In addition, the following initial and boundary conditions apply:

(9) For t=0, 0≤x≤L: c=0 

(10) $$\mathrm{For}\;t\geq 0,\;x=0:-{\;D}_{\mathrm{eff}}\times \frac{\partial c}{\partial x}=v_{\mathrm{eff}}\times \left(c_{0}-c\right)$$

(11) $$\mathrm{For}\;t\geq 0,\;x=L:\;\frac{\partial c}{\partial x}=0,$$

where:

- $c_{0}$: Concentration of the NaCl ions in the NaCl solution (mol/m³);
- L: Length/depth of the probe (m).

Different approaches for obtaining analytical and numerical solutions to the problem were derived, each with different advantages and disadvantages, e.g., regarding the simplification of the problem, the complexity of implementing the solution, and the computational load. They are discussed in the following sections.

While Section 2.2 and Section 2.3 refer to an analytical and efficient numerical solution approach of the simplified 1D-problem assuming one material only, Section 2.4 deals with a more complex numerical solution approach for the problem in 3D assuming a multi-material geometry.

### 2.2. Analytical Solution of the Simplified 1D-Problem

Bastian and Lapidus [10] provide the basis for an approach to analytically solve the system of equations. First, the variables are changed as follows:

(12) $$c-c_{0}=w\times e^{\frac{v_{\mathrm{eff}\;}\times \;x}{2\;\times \;D_{\mathrm{eff}}}-\frac{v_{\mathrm{eff}}^{2}\;\times \;t}{4\;\times \;D_{\mathrm{eff}}}}$$

with this, as well as with

(13) $$h=\frac{v_{\mathrm{eff}}}{2\times D_{\mathrm{eff}}}$$

where h: Film coefficient (m).

Equations (6) and (9)–(11) can be simplified according to Dankwerts [11]:

(14) $$D_{\mathrm{eff}}\times \frac{\partial^{2}w}{\partial x^{2}}=\frac{\partial w}{\partial t}$$

(15) $$\mathrm{For}\;t=0,\;0\leq x\leq L:\;w=-c_{0}\times e^{-h\;\times \;x}\;$$

(16) $$\mathrm{For}\;t\geq 0,\;x=0:\;\frac{\partial w}{\partial x}=h\times w$$

(17) $$\mathrm{For}\;t\geq 0,\;x=L:\;\frac{\partial w}{\partial x}=-h\times w$$

according to Carslaw and Jaeger [12], the transformed solution then looks like:

(18) $$w=2\times \overset{\infty }{\underset{n=1}{\sum }}e^{-D_{\mathrm{eff}}\times \alpha_{n}^{2}\times t}\times \frac{\alpha_{n}\times \cos\alpha_{n}\times x+h\times \sin\alpha_{n}\times x}{\left(\alpha_{n}^{2}+h^{2}\right)\times L+2\times h}\times I,$$

where

(19) $$I=\int_{0}^{L}-c_{0}\times e^{-h\times x}\times (\alpha_{n}\times \cos\alpha_{n}\times x+h\times \sin\alpha_{n}\times x)\mathrm{dx}$$

based upon Equations (18) and (19), Bastian and Lapidus [10] formulated the solution for the concentration at x = L, and Mohsen et al. [13] formulated the solution for different boundary conditions. The integral in Equation (19) is solved to give for 0 < x ≤ L considering Equations (15)–(17):

(20) $$-\frac{I}{c_{0}}=\alpha_{n}\times {\left(\frac{e^{-h\times x}}{\alpha_{n}^{2}+h^{2}}\times \left(-h\times \cos\alpha_{n}\times x+\alpha_{n}\times \sin\alpha_{n}\times x\right)\right)}_{0}^{L}$$

this finally results in:

(21) $$-\frac{I}{c_{0}}=\frac{e^{-h\times L}}{\alpha_{n}^{2}+h^{2}}\times \left(-2\times \alpha_{n}\times h\times \cos\alpha_{n}\times L+\left(\alpha_{n}^{2}-h^{2}\right)\times \sin\alpha_{n}\times L\right)+\frac{2\times \alpha_{n}\times h}{\alpha_{n}^{2}+h^{2}},$$

where, according to Carslaw and Jaeger [12], $\alpha_{n}$ represents the roots of:

(22) $$\tan\alpha_{n}\times L=\frac{2\times \alpha_{n}\times h}{\alpha_{n}^{2}-h^{2}}$$

Equation (22) can be transformed to:

(23) $$\frac{2\times h}{\tan\alpha_{n}\times L}=\alpha_{n}-\frac{h^{2}}{\alpha_{n}},\;$$

with the substitution

(24) $$\alpha_{n}\times L=r_{n}$$

Equation (23) finally becomes:

(25) $$2\times \cot r_{n}=\frac{r_{n}}{h\times L}-\frac{h\times L}{r_{n}}$$

according to Carslaw and Jaeger [12], the positive roots can be found in any interval (n × π, (n + 1) × π), for n ∈ N. This allows us to calculate w according to Equation (18) and, finally, taking into account the change in variables according to Equation (12), to calculate the time- and depth-dependent solution for the concentration c. In Equation (18), the infinite sum is approximated by a finite sum using the first n terms.

### 2.3. Numerical Solution with Finite Differences of the Simplified 1D-Problem

The finite difference method is an established method for solving differential equations based on the discretization of derivatives [14,15]. Using the finite difference method, Equation (6) can be discretized for the numerical solution on grid points in a two-dimensional time–space grid, and is represented as follows:

(26) $$\frac{c_{i}^{j+1}-c_{i}^{j}}{\mathrm{dt}}=D_{\mathrm{eff}}\times \frac{\left(c_{i}^{j}-2\times c_{i}^{j}+c_{i-1}^{j}\right)}{{\mathrm{dx}}^{2}}-v_{\mathrm{eff}}\times \frac{\left(c_{i+1}^{j}-c_{i-1}^{j}\right)}{2\times \mathrm{dx}},$$

where $c_{i}^{j}=c\left(t=j\times \mathrm{dt},x=i\times \mathrm{dx}\right)$, and

- $c_{i}^{j}$: Discretized solution for the concentration (mol/m³);
- j: Time index in solution grid, j ∈ $N_{0}$, j = $0\ldots n_{t};$
- i: Spatial index in solution grid, i ∈ $N_{0}$, i = $0\ldots n_{x};$
- $n_{x}$: Maximum spatial index in solution grid, $n_{x}\in N$;
- $n_{t}$: Maximum time index in solution grid, $n_{\;t\;}\in N$;
- dt: Time increment in solution grid (t);
- dx: Spatial increment in solution grids (m).

For the initial and boundary conditions, the following holds:

(27) $$\mathrm{For}\;j=0,\;i=0\ldots n_{x}:{\;c}_{i}=0\;$$

(28) $$\mathrm{For}\;j=0\ldots n_{t}:-D_{\mathrm{eff}}\times \frac{\left(c_{1}^{j}-c_{0}^{j}\right)}{\mathrm{dx}}=v_{\mathrm{eff}}\times \left(c_{0}-c_{0}^{j}\right)$$

(29) $$\mathrm{For}\;j=0\ldots n_{t},\;i=n_{x}:\;\frac{c_{i+1}-c_{i}}{\mathrm{dx}}=0\;$$

Equation (26) is transformed to determine the concentration. For area I, the result for the water-saturated case with convection due to migration only and after introducing a fitting factor for the migration speed is:

(30) $$c_{i}^{j+1}=c_{i}^{j}+D_{\mathrm{eff}}\times \frac{\mathrm{dt}}{{\mathrm{dx}}^{2}}\times \left(c_{i+1}^{j}-2\times c_{i}^{j}+c_{i-1}^{j}\right)-f_{\mathrm{net}}\times v\times \frac{\mathrm{dt}}{2\times \mathrm{dx}}\times \left(c_{i+1}^{j}-c_{i-1}^{j}\right),$$

where $f_{\mathrm{net}}$: Fitting factor for the migration speed.

Assuming an electric field exclusively in area II, $f_{\mathrm{net}\;}$= 0 applies in Equation (30). However, the fitting factor can also be used later to model a weakened effect of the electric field in area I. The latter can be observed in the results from the finite elements method (FEM) simulations discussed in the following section of this paper. In this context, area I can be divided into subareas with different fitting factor values for an approximation of this effect.

For area II, the following applies:

(31) $$c_{i}^{j+1}=c_{i}^{j}+D_{\mathrm{eff}}\times \frac{\mathrm{dt}}{{\mathrm{dx}}^{2}}\times \left(c_{i+1}^{j}-2{*c}_{i}^{j}+c_{i-1}^{j}\right)+v\;\times \frac{\mathrm{dt}}{2*\mathrm{dx}}*\left(c_{i+1}^{j}-c_{i-1}^{j}\right)$$

for j =0…nt, i = nt, the following applies:

(32) $$c_{i}^{j+1}=c_{i}^{j}+D_{\mathrm{eff}}\times \frac{\mathrm{dt}}{{\mathrm{dx}}^{2}}\times \left(c_{i}^{j}-2{*c}_{i}^{j}+c_{i-1}^{j}\right)+v_{\mathrm{eff}}*\frac{\mathrm{dt}}{\mathrm{dx}}\times \left(c_{i}^{j}-c_{i-1}^{j}\right)$$

for $j\;=0\ldots n_{t}$, the following applies:

(33) $$c_{0}^{t}=\frac{\left(v_{\mathrm{eff}}\times {\;c}_{0}+\frac{D_{\mathrm{eff}}}{\mathrm{dx}}\times {\;c}_{1}^{t}\right)}{v_{\mathrm{eff}}+\frac{D_{\mathrm{eff}}}{\mathrm{dx}}}$$

thus, the discretized concentration $c_{i}^{t}$ can be determined starting from Equation (27) for t = 0 as well as from Equations (30)–(33) for all following time steps for all grid points. It is to be iterated over all time and location indices, i.e., $c_{i}^{j+1}$ is calculated for j = $0\ldots (n_{t}-$1) and $i\;=1\ldots n_{x}$.

### 2.4. Solution with the Finite Element Method for the 3D Problem with Multi-Material Geometry

Using the finite element method (FEM), modern FEM multiphysics simulation software like COMSOL is able to solve transient problems arising from differential equations for almost arbitrarily complex geometries in 3D space, and potentially consider multiple materials as well as physical phenomena at the same time. COMSOL is proprietary and commercially available. It is provided by COMSOL AB [16,17,18].

**Figure 3:**
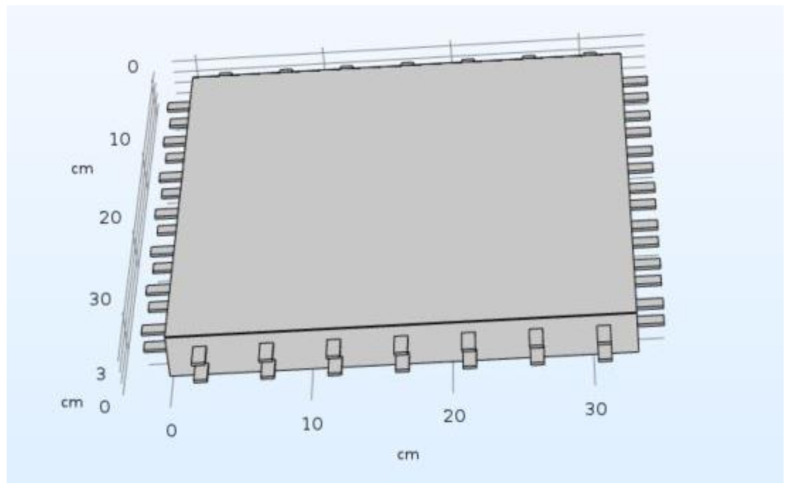
3D model of the multi-material geometry for the FEM simulation with COMSOL.

To calculate the ingress of NaCl ions into the probe, the simulation software COMSOL was used. In COMSOL, the problem was not modelled in 1D but in 3D, based on a specifically designed multi-material geometry resembling the actual bridge construction as much as possible, as shown in Figure 3.

The model was designed as a multi-material 3D geometry of two carbon meshes included in a block of mortar. Within the simulation, in between the two carbon layers, a difference in the electrical potential is defined that automatically leads to the generation of realistic electrical field lines within and around the layers, which influences the flow of chloride ions.

As an initial condition, the concentration of NaCl within the probe was defined to be zero, while, as a boundary condition, a 4% NaCl solution was applied to the top of the probe, leading to a continuous ingress over time.

In COMSOL, the modules “AC/DC” and “Chemical Reaction Engineering” were used for the simulation of the NaCl ingress into the probe. In this way, the transient and 3-dimensional behavior of the NaCl ions due to diffusion and migration could be considered. Since the probes are assumed to be water-saturated, convection due to water transport into the probe was neglected. Therefore, the mathematical problem solved with COMSOL, for a variety of distances and different levels of potential in the carbon layers, goes beyond the one described in Equations (1)–(11) and more accurately resembles the actual set-up due to the consideration of 3-dimensionality, electrical field lines, and the materialistic existence of the physical carbon layers.

During the modelling, the two problem types, “Electrical Currents” and “Transport of Diluted Species”, were added and configured for the relevant coupled simulation.

For “Electrical Currents” the default set of equations for the stationary case was selected with the default value of 50 ohm for the reference impedance, whereas for the “Transport of Diluted Species”, Equations (6)–(11) were adopted and given for the 3D-case. Only migration in the electrical field was activated as transport mechanism for which the Nernst–Einstein relationship was selected as an option in COMSOL for modelling.

For both problems, in COMSOL, the physics-controlled meshing option was activated including free tetrahedron meshes. The size of the elements was configured to be “extra-fine” resulting in a minimum and maximum element size of 0.0555 cm and 1.29 cm, respectively.

For the solution, quadratic shape functions were chosen for the “Electrical Currents” and linear ones for the “Transport of Diluted Species”.

Table 1 shows the relevant parameter values configured for the multi-physics simulation.

## 3. Results—Evaluation and Visualization of the Solutions

Based upon the mathematical approaches introduced, a variety of solutions for different parameter configurations were calculated for visualization and comparisons. The comparisons serve two purposes: First, assuring a rough consistency between the solutions for the same parameter configurations as an indicator of valid implementations; second, investigating specific behavior of the NaCl concentration in several solution approaches, i.e., the behavior of NaCl concentrations over different migration configurations at certain depths of the probe. Differences in the various approaches to obtaining a solution are identified and discussed.

### 3.1. Comparison of Analytical and Finite Differences Solutions

The analytical solution was calculated using a symbolic Matlab script, which was also used for the determination of the positive roots in Equation (25), for the following parameters over a time period of 1 s, see Table 2. Note that the units here refer to parameters integrated over the cross-section area of the specimen due to the assumed 1-dimensionality of the problem.

The parameters chosen here do not yet correspond to realistic values of the observed use-case. In particular, the choice of a significantly increased diffusion coefficient, which leads to a correspondingly high ion flux, is intended to reveal irregularities in the calculated solution as well as differences between the analytical and numerical solutions.

**Figure 4:**
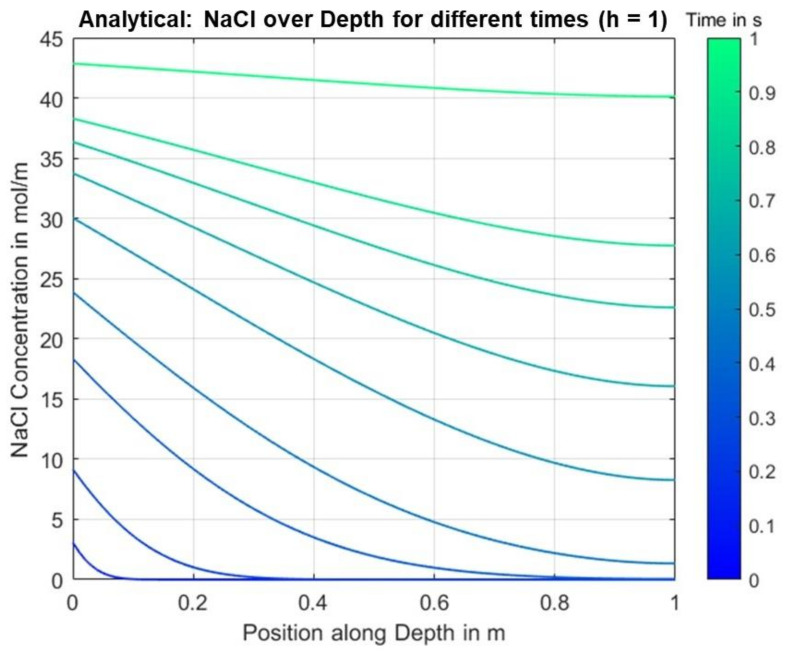
Analytical solution results for the concentration over position for different times.

Figure 4 shows the calculated NaCl concentration along the depth for different time points.

**Figure 5:**
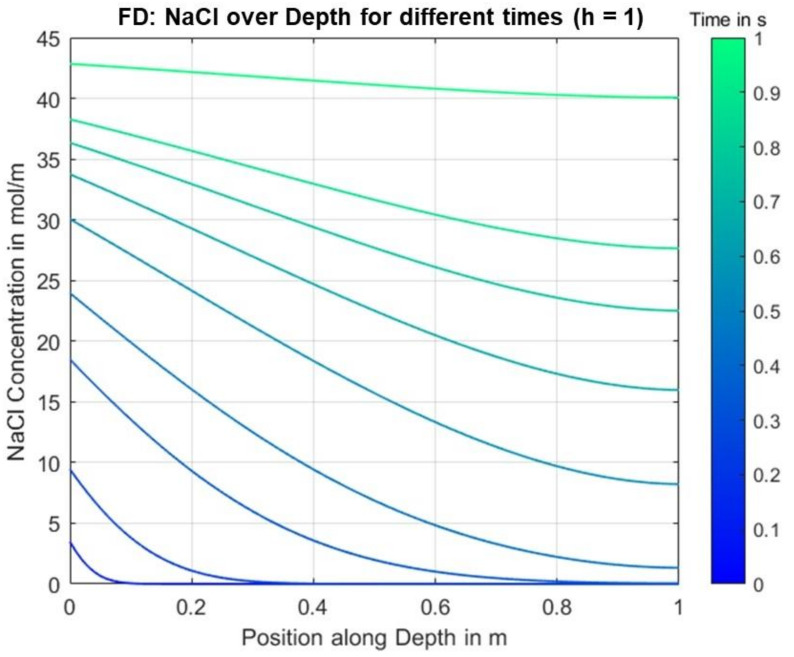
Finite differences solution results for the concentration over position for different times.

Figure 5 shows the NaCl concentration calculated and visualized accordingly based on the finite differences solution with only area II being considered for reasons of comparability.

It can be seen that despite the high ion fluxes, there are no significant differences, especially for later time points. Thus, it can be deduced that the numerical method generally provides a very well approximated solution with negligible errors for, at least, an equally fine discretization, i.e., identical or smaller values for dt and dx, for smaller fluxes due to lower, more realistic diffusion coefficients.

The finite differences solution offers the decisive advantage that, in contrast to the analytical solution, the different migration directions in areas I and II can be taken into account.

In addition, weakly pronounced field lines, as can be seen later in the results of the FEM simulation, can be considered in area I.

The finite differences solutions were calculated for both areas over a total time period of 410 d for the parameter values shown in Table 3. A weakened effect of the electrical field in area I was considered using fitting factors for the migration speed, cf. Equation (30).

Through the fitting factor in Equation (30), the effect of the electric field at different depth ranges was modeled as follows:

(34) $$f_{\mathrm{net}}=\left\{\begin{array}{c} 0.1\times min{\left(h,1\right)}^{2}for\;x\;<0.005m\\ 0.2\times min{\left(h,1\right)}^{2}\;for\;x\;<0.010m\\ 0for\;x\;>0.025\;m\\ 1,\;else \end{array}\right.$$

whereas the reasoning for the last two fitting factor values is obvious, the first two values here (x < 0.005 m and x < 0.010 m) are the results of an optimization aiming for maximum similar values of the resulting concentrations as they were observed in the experiments.

**Figure 6:**
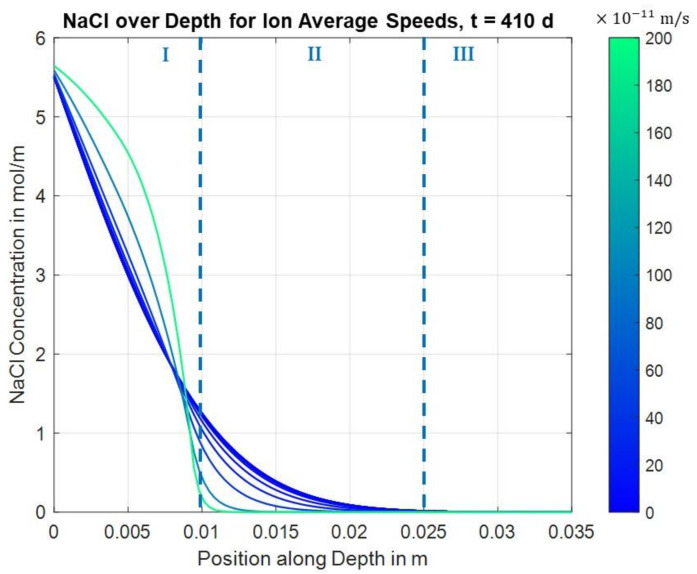
Finite differences solutions for concentration over position for different migration velocities (t = 410 d).

Figure 6 shows the calculated concentration for different migration velocities along the depth. It can clearly be seen that higher migration velocities partly lead to higher concentrations in the initial area and to lower concentrations in deeper areas.

**Figure 7:**
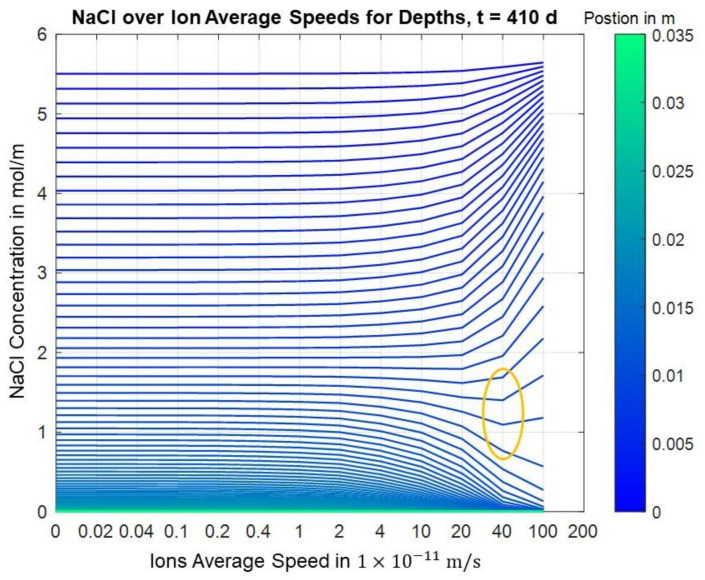
Finite differences solution for concentration over migration speed for different positions (t = 410 d).

The effect of the migration speed on the concentration at different depths is clearly shown in the following image for t = 410 d. The trends for higher migration velocities, of higher concentrations in the lower depths and lower concentrations in deeper areas, which were indicated by the previous figure, are confirmed. Moreover, it can be seen that there is an effect in area I slightly above the first layer where a local minimum is formed. Here, higher migration velocities initially lead to lower concentrations before they lead to higher concentrations again. However, it is also clear that in the deeper layers, i.e., at positions in area II below the first carbon layer, higher migration velocities always lead to lower concentrations, which is the expected effect of the chloride barrier. The transition from area I to area II is marked in Figure 7.

The behavior described can also be seen in the following figures, which take into account the dimension of time and thus also consider the time dependency of the concentration versus the migration speed.

**Figure 8:**
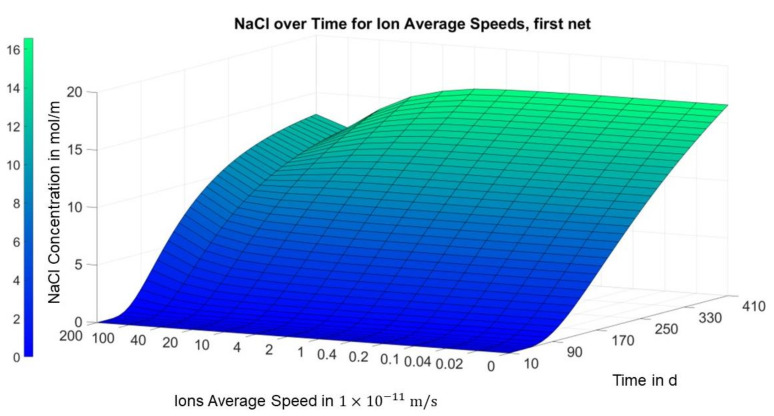
Finite differences solution for concentration over migration speed and time (position = 0.008 m; area I, slightly above the first carbon mesh).

**Figure 9:**
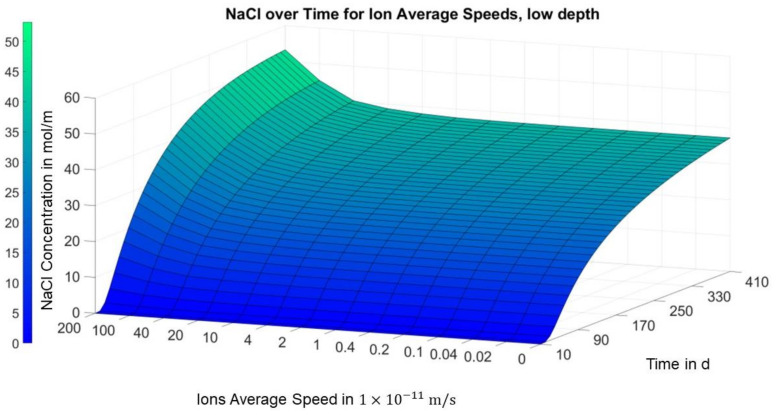
Finite differences solution for concentration over migration speed and time (position = 0.008 m; area I, slightly above the first carbon mesh).

Figure 8 illustrates that the aforementioned local minimum of the concentration over the migration speed slightly above the first carbon layer in area I only forms after a few months. In Figure 9, on the other hand, a consistent positive trend can be seen in the concentration over the migration speed in the middle of area I for all time points.

**Figure 10:**
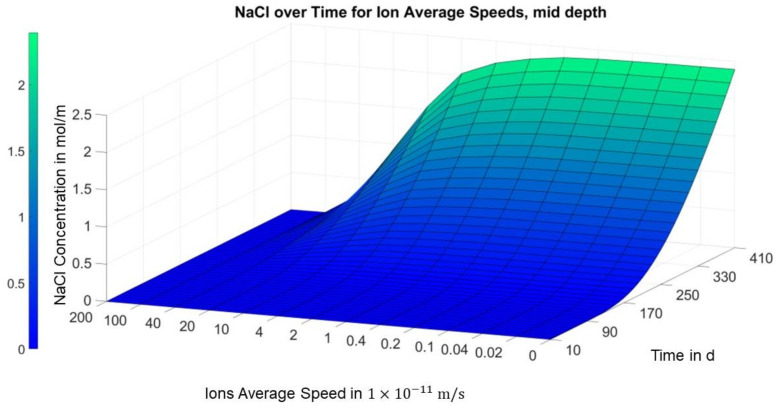
Finite differences solution for concentration over migration speed and time.

Figure 10 shows the concentration curve in area II in the middle of the sample. Here, in contrast to the previous image, the concentration decreases steadily over the migration speed for all the time points.

**Figure 11:**
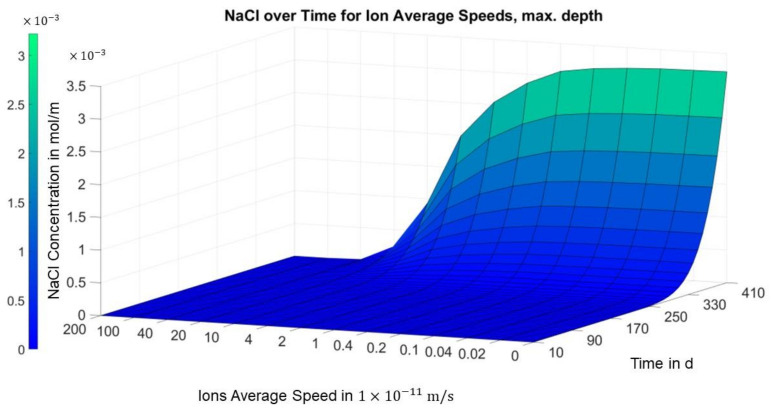
Finite differences solution for concentration over migration speed and time (position = 0.035 m, area III, bottom).

In Figure 11, referring to the bottom of the specimen, it can be seen that the concentration curve predictably also decreases steadily for all time points. After 410 days, the concentration for the two highest migration velocities at the end of the sample is still close to 0.

The temporal gradient of the concentration also increases strongly for the smallest migration velocities, i.e., stationarity is still far from being reached after 410 days.

### 3.2. Visualization and Discussion of the FEM Solution

Based on the parameter configuration and the initial and boundary conditions as described above, a variety of simulations was performed for different potential differences between the carbon layers ranging from 0 to 3 V.

**Figure 12:**
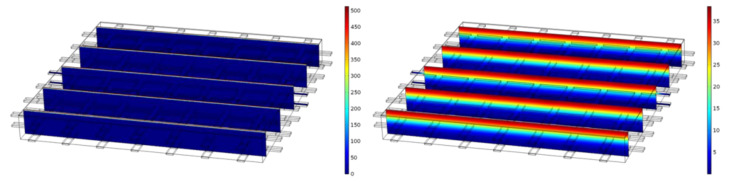
A 3D model of the FEM solution for the NaCl concentration for 0 V at t = 0 d (left) and t = 650 d (right).

Figure 12 shows the distribution of the NaCl concentration along the depth without an electrical field, a case serving here as a reference for the other non-zero cases. As expected, for t = 650 d, a continuous and smooth change of the NaCl concentration along the depth can be seen, which mainly seems to be 1-dimensional due to the constant distribution at the same depth along the other directions.

**Figure 13:**
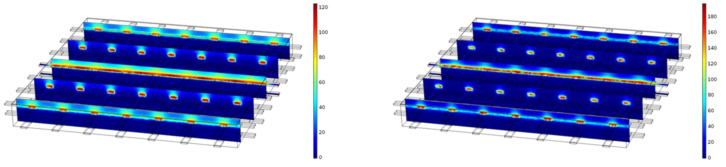
A 3D model of FEM solution for the NaCl concentration at t = 650 d for 0.5 V (left) and 1 V (right).

Compared to Figure 12, Figure 13 illustrates a significantly different concentration distribution for the same t = 650 d due to the applied potential differences of 0.5 V and 1 V. For both cases, a clear dependency can also be seen on the other directions. The concentration of NaCl is much higher at and around the carbon meshes. A continuous and smooth change of the NaCl concentration in the case of 0 V cannot be observed anymore. The maximum concentration values even seem to be higher than in Figure 12.

Apart from that, for the higher voltage of 1 V the maximum concentration is higher than for the case of 0.5 V.

An observation that can be made in Figure 13 is an increased concentration above the first carbon layer for both cases, with the case of 1 V showing higher concentrations at these locations than the case of 0.5 V, contradicting the first expectation that the electrical field would exist only between the two carbon layers.

**Figure 14:**
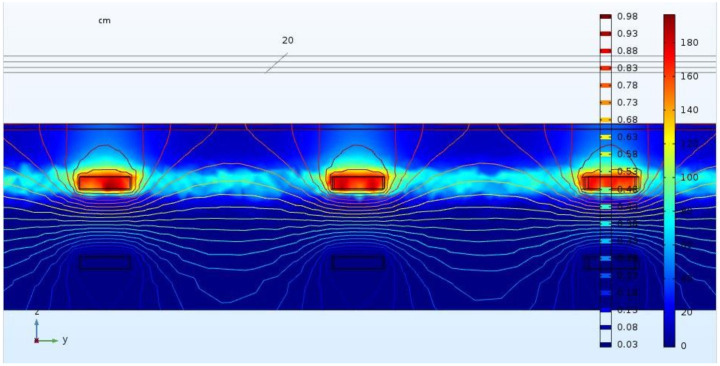
A 2D cut of the FEM solution for the NaCl concentration at 1 V and t = 650 d showing potential lines.

Figure 14 provides the reason for this effect; the rounded shape of the potential lines also reaching depths above the first and below the second carbon layer. The qualitative course of the potential lines does not change with changing voltages. Furthermore, a shift in one carbon layer of about a half the size of a mesh does not lead to a significant change in the chloride concentrations.

**Figure 15:**
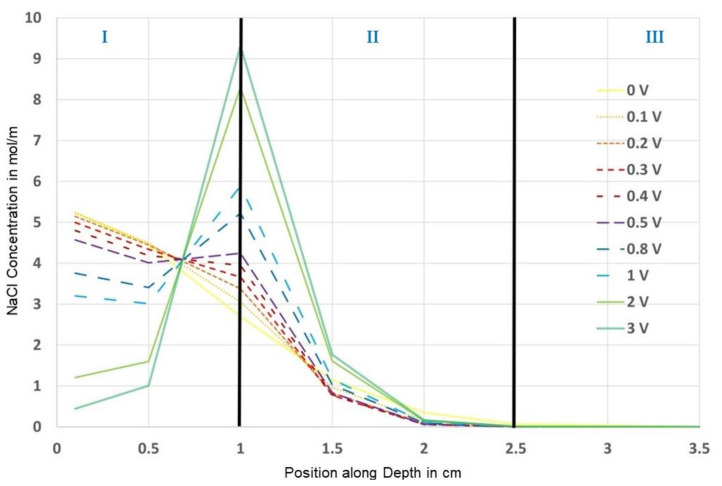
FEM solutions for the NaCl concentration over depth for different voltages at t = 410 d.

The resulting concentration over the depth of the probe at t = 410 d is plotted in Figure 15.

It can be observed that higher voltages lead to higher concentrations at certain positions, especially around the first carbon layer, i.e., the higher the voltage, the higher the concentration.

However, this effect is reversed the more you transition from the first carbon layer to the top or to the bottom, because the chloride ions are either dragged more into the probe from the top or are held back more from diffusing into the depth, respectively, the higher the voltage is. It can be seen in Figure 15 that from a position slightly above the depth of the second layer, all voltages provide a lower concentration than the reference, which represents the desired chloride barrier effect.

**Figure 16:**
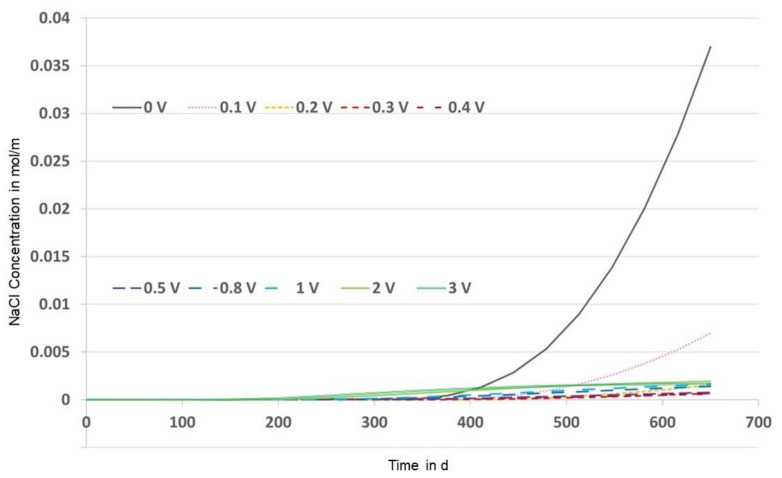
FEM solutions for the NaCl concentration over time at x = L (area III bottom).

To elucidate this effect, and due to the additional time dependency, the NaCl concentration is shown in Figure 16 at the bottom of the probe over time up to 650 d. It becomes apparent that t = 410 d approximately marks the time point from which all voltages provide a lower concentration than the reference.

Otherwise, it seems that the curves of lower voltages are based on higher gradients over time than those of higher voltages, i.e., the longer one waits, the lower the concentration at the bottom will be for higher voltages compared to lower voltages. Here, for t = 410 d, the concentrations at 2 V and 3 V are not the lowest; the lowest is the concentration at 0.3 V.

## 4. Discussion—Overall Solution Comparison and Evaluation

**Figure 17:**
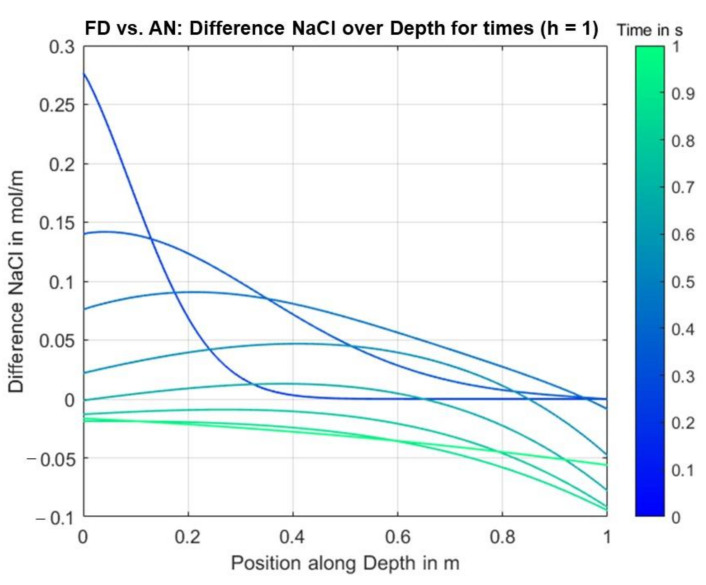
Differences between concentrations from analytical and finite differences solution.

To compare the analytical and finite differences solution approach, differences between the solution values were evaluated over time and position along the depth of the specimen by subtracting the analytical solution values from the ones from the finite differences solution. Figure 17 shows the differences between the concentration curves for identical parameter values, cf. Table 2. Only area II of the specimen was considered.

It can be seen that there are no significant differences, especially for later time points. Thus, it can be deduced that the numerical method generally provides a very well approximated solution of the simplified 1D problem for, at least, an equally fine discretization, i.e., identical or smaller values for dt and dx, for equal or smaller fluxes.

However, in the course of the discussion of the analytical and finite differences solution, it became apparent that the results of the analytical solution can primarily only serve for checking the validity of the solution values obtained by the implementation of the finite differences method for a specific set-up without changing migration directions over the depth of the probe. Even though it is possible with the help of the analytical solution to model migration acting against diffusion, the missing change of the migration directions and, therefore, an incorrect boundary condition at the top of the probe, render the analytical solution too simplified and thus unrealistic.

The finite differences model, by contrast, is able to consider changing migration directions along the depth with the correct boundary condition at the top. A fitting factor was introduced for the finite differences model (Equations (30) and (34)), to consider a weakened effect of the electrical field above the depth of the first carbon layer.

The relevant solutions were investigated thoroughly over position and time for different migration velocities.

The FEM solution was finally used not only to solve the simplified mathematical–physical problem in 1D, but also to obtain a model as close as possible to reality, i.e., considering a multi-material 3D geometry in addition to the exact shapes of the electrical field lines. This led to solutions showing 3D distributions of the NaCl concentrations over time. It could be seen that the application of potential differences leads to dependencies on more than one spatial dimension.

**Figure 18:**
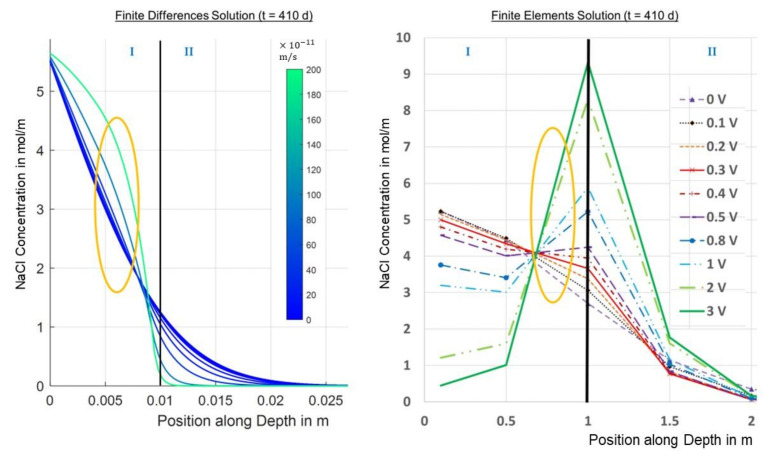
Investigated effect in the finite differences and the finite elements solution for t = 410 d.

From both the finite differences and the FEM solutions, it could be seen that higher migration velocities or voltages, respectively, lead to higher concentrations in an area slightly above the first carbon layer, whereas lower concentrations appear for higher migration velocities or voltages, respectively, the more you move to the bottom of the probe, which is the intended effect of the electrochemical chloride barrier; see Figure 18.

The finite differences solution did not show decreasing concentrations at the very top of the specimen for increasing voltages, in contrast to the FEM solution. Furthermore, for the FEM solution, the curves for the higher voltages cut the curves for the lower voltages below the first carbon layer in area II, in contrast to the finite differences solution, where this takes place above the first carbon layer. The reason for that might be the mathematical limitations of the finite differences method or the underlying simplification of the mathematical–physical problem. However, the experimental investigations showed the cut below the first carbon layer in accordance with the FEM solution [3].

**Figure 19:**
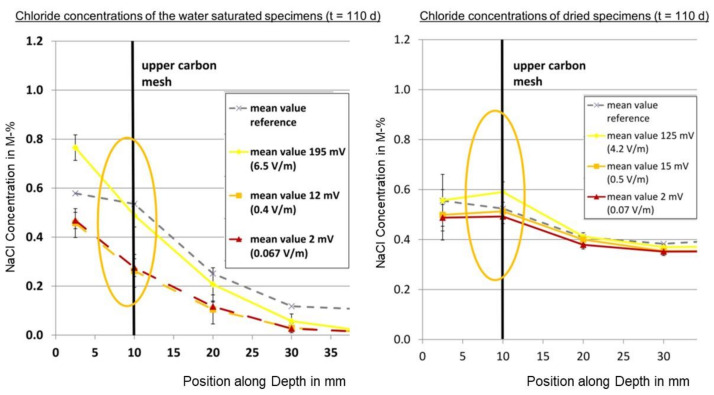
Investigated effect in the experimentally determined chloride concentration for t = 110 d.

The unexpected effect of higher chloride concentrations for higher voltages or electrical fields of the chloride barrier, respectively, which was observed in the course of the experimental investigations, appears slightly above the first carbon layer for both the finite differences and the finite elements solution; cf. Figure 18 and Figure 19.

Figure 8 shows a local minimum occurring after a certain amount of time, i.e., the chloride concentration decreases with the migration speed up to a certain migration speed before it increases again. This explains the unexpected observation in the experimental and model-based investigations only by acting diffusion and migration.

In summary, the magnitude of the order of the chloride concentration values calculated with the finite differences and FEM solutions, as well as the aforementioned effects, are consistent with the ones observed in experimental investigations that provided the motivation for this paper.

## 5. Conclusions

Higher chloride concentrations for stronger electrical fields, as well as a local minimum of the chloride concentrations over the strength of the electrical field or the migration speed, respectively, were observed for one duration for certain depths in test specimens with an electrochemical chloride barrier in experiments preceding this work [3]. These effects were unexpected, could not be explained in the first place, and were, therefore, analytically and numerically investigated in this paper. The following conclusion points can be deduced based upon the results discussed:

- The effects found in the experiments can be confirmed by the developed and presented finite differences model and the performed FEM simulations, and are explainable solely by the acting diffusion and migration in the test specimens;
- While the performed FEM simulations modelled the chloride flow the closest to reality and also revealed the weakened electrical migration above the first carbon layer, the finite differences model also showed the investigated effects above the first carbon layer;
- The effect of the local minimum is limited to positions above the depth of the first carbon layer of the test specimens. It is caused by the electrical field between the first and second carbon layer also generating a weakened electrical migration directed into the test specimen slightly above the first carbon layer;
- According to the experimental results and the ones from the FEM simulation, higher migration velocities or stronger electrical fields, respectively, lead to lower chloride concentrations for all positions below the first carbon layer only after sufficient time duration. Therefore, the intended effect of an electrochemical chloride barrier can in general only be observed and confirmed after a certain time depending on conditions and material parameters. FEM can help to approximate this change point.

## Figures and Tables

**Table 1:** Parameter values used in COMSOL for the multi-physics simulation.

Parameter	Value	Unit
Length/Width/Height of mortar specimen	0.3318/0.3318/0.035	m
Spacing of the carbon meshes	0.038	m
Length/Width/Height of carbon layers	0.3318/0.3318/0.00225	m
Depths of the carbon meshes from top	0.01/0.025	m
Shape Function “Electrical Currents”	Quadratic	-
Shape Function “Transport of Diluted Species”	Linear	-
Discretization Method	Physics-controlled	-
Mesh Type	Tetrahedron	-
Min./Max. Element Size	0.0555/1.29	cm
Curvature Factor	0.3	-
Solver (both problems)	Linear iterative	-
Nonlinear method for damping (both problems)	Newton (constant)	-
Termination Technique	Tolerance	-
Termination Tolerance	1	-
Total number of Degrees of Freedom	560,989	-

**Table 2:** Parameter values used for the visualized analytical solution.

Parameter	Value	Unit
c_0_	44.44	mol/m
L	1	m
D_eff_	1	1/s
h	1	m

**Table 3:** Parameter values used for the visualized finite differences solutions.

Parameter	Value	Unit
c_0_	63.25	mol/m
L	0.035	m
D_eff_	$1\times 10^{-12}$	1/s
h	0, 0.1, 0.2, 0.5, 1, 2, 5, 10, 20, 50, 100, 200, 500, 1000	m
dt	886	s
dx	0.00035	m
L_net1_	0.01	m
L_net2_	0.025	m

L_net1_: Depth of the first net of the E-field (m); L_net2_: Depth of the second net of the E-field (m).

## Data Availability

Can be provided upon reasonable request.
